# Cytologically Suggested Vulvar Involvement in a Dog with Multicentric Lymphoma After a Nodal Response to CHOP-Based Chemotherapy, with Discordant Clonality Results: A Case Report

**DOI:** 10.3390/vetsci13101102

**Published:** 2026-10-16

**Authors:** Taehyun Roh, Kunho Song

**Affiliations:** 1 Department of Veterinary Internal Medicine, College of Veterinary Medicine, Chungnam National University, Daejeon 34134, Republic of Korea; freegith@gmail.com; 2 Soop Animal Medical Center, Daejeon 35233, Republic of Korea

**Keywords:** canine, lymphoma, vulva, disease progression, clonality, PCR for antigen receptor rearrangement

## Abstract

Involvement of the female reproductive tract is rare in canine lymphoma. A 9-year-old spayed female Maltese dog was presented with generalized lymphadenopathy. Cytology of a mandibular lymph node aspirate indicated large cell lymphoma, whereas PCR for antigen receptor rearrangement (PARR) of the same sample was polyclonal. Splenectomy confirmed splenic large cell lymphoma, classified as World Health Organization clinical stage IVa. After 12 cycles of a CHOP (cyclophosphamide, doxorubicin, vincristine, and prednisolone)-based protocol, restaging computed tomography showed a marked reduction in all measurable lymph nodes, whereas gastric and subcutaneous nodules persisted. On Day 181 (168 days after the start of chemotherapy), vulvar swelling was noticed and progressed despite antimicrobial and anti-inflammatory treatment, together with inguinal lymphadenopathy. Disease progression was diagnosed by inguinal lymph node cytology, and rescue chemotherapy was started; however, the dog developed acute dyspnea and died two days later. Results reported after death showed a mixed monoclonal and polyclonal immunoglobulin heavy chain (IgH) pattern in the inguinal lymph node and a clonal IgH rearrangement in the vulvar aspirate, which was cytologically consistent with lymphoma. In dogs treated for lymphoma, vulvar swelling may reflect neoplastic infiltration, and PARR results may differ between samples obtained at different sites and time points.

## Simple Summary

Lymphoma is a common cancer of the immune system in dogs, but it rarely affects the female reproductive organs. This report describes a 9-year-old Maltese dog with lymphoma involving multiple lymph nodes and the spleen. After the spleen was removed to confirm the diagnosis, the dog received a standard combination chemotherapy protocol, and all measured lymph nodes became much smaller. About five and a half months after chemotherapy began, the vulva became swollen and did not improve with antibiotics or anti-inflammatory drugs. A needle sample from the vulva later suggested lymphoma. A genetic test used to support the diagnosis gave different results depending on where and when the sample was taken. This case shows that vulvar swelling in a dog treated for lymphoma may be caused by the cancer itself, and that an unclear result of this test should not be used on its own to rule out the disease.

## 1. Introduction

Lymphoma is one of the most common hematopoietic neoplasms in dogs, and the multicentric form accounts for the majority of cases [1,2]. Apart from the alimentary and cutaneous forms, lymphoma arising at miscellaneous extranodal sites represents approximately 1% of canine lymphomas [3]. Involvement of the female reproductive tract has rarely been documented; published cases have mainly involved the uterus or ovaries, including a primary marginal zone B-cell lymphoma of the uterine horn and a T-cell lymphoma infiltrating both the uterus and ovaries [3,4].

Mucocutaneous junctions, including the vulva, are frequently affected in canine cutaneous epitheliotropic T-cell lymphoma [5,6]. That condition, however, is a primary cutaneous T-cell disease and differs from involvement of a mucocutaneous site by multicentric lymphoma. Non-epitheliotropic cutaneous B-cell lymphoma has also been reported, but it is uncommon [7]. In a search of the literature, the authors did not find a report of vulvar involvement detected during follow-up of a dog treated for multicentric lymphoma.

Polymerase chain reaction (PCR) for antigen receptor rearrangement (PARR) is widely used to support the diagnosis of lymphoid neoplasia, with a reported sensitivity of approximately 86–91% [8,9]. False-negative results can occur when the consensus primers fail to amplify the rearranged gene of the neoplastic clone, for example because of somatic hypermutation in B-cell neoplasms, or when a clonal population is masked by a large number of non-neoplastic lymphocytes [8,9,10]. In addition, PARR does not reliably establish cell lineage, because cross-lineage rearrangements may occur [10]. Clonality results should therefore be interpreted together with cytology, histopathology, and immunophenotyping [10].

This report describes a dog with multicentric lymphoma that showed a marked nodal response to a CHOP (cyclophosphamide, doxorubicin, vincristine, and prednisolone)-based protocol and in which vulvar swelling was the first sign of disease progression noticed by the owner. PARR results differed between samples obtained at different sites and time points, and the possible reasons for these discrepancies are discussed.

## 2. Case Presentation

### 2.1. Signalment, History, and Presentation

A 9-year-old spayed female Maltese dog (2.5 kg) was presented with cervical swelling and enlarged mandibular lymph nodes (Day 0). Cardiac disease had been diagnosed at another hospital 2–3 years earlier, and the dog was receiving pimobendan and a renin–angiotensin–aldosterone system inhibitor, together with medication for chronic bronchial disease. Physical examination revealed generalized peripheral lymphadenopathy, most prominent in the right mandibular lymph node. No systemic signs were reported by the owner. The main clinical events are summarized in Table 1.

### 2.2. Clinicopathological and Imaging Findings

**Figure 1:**
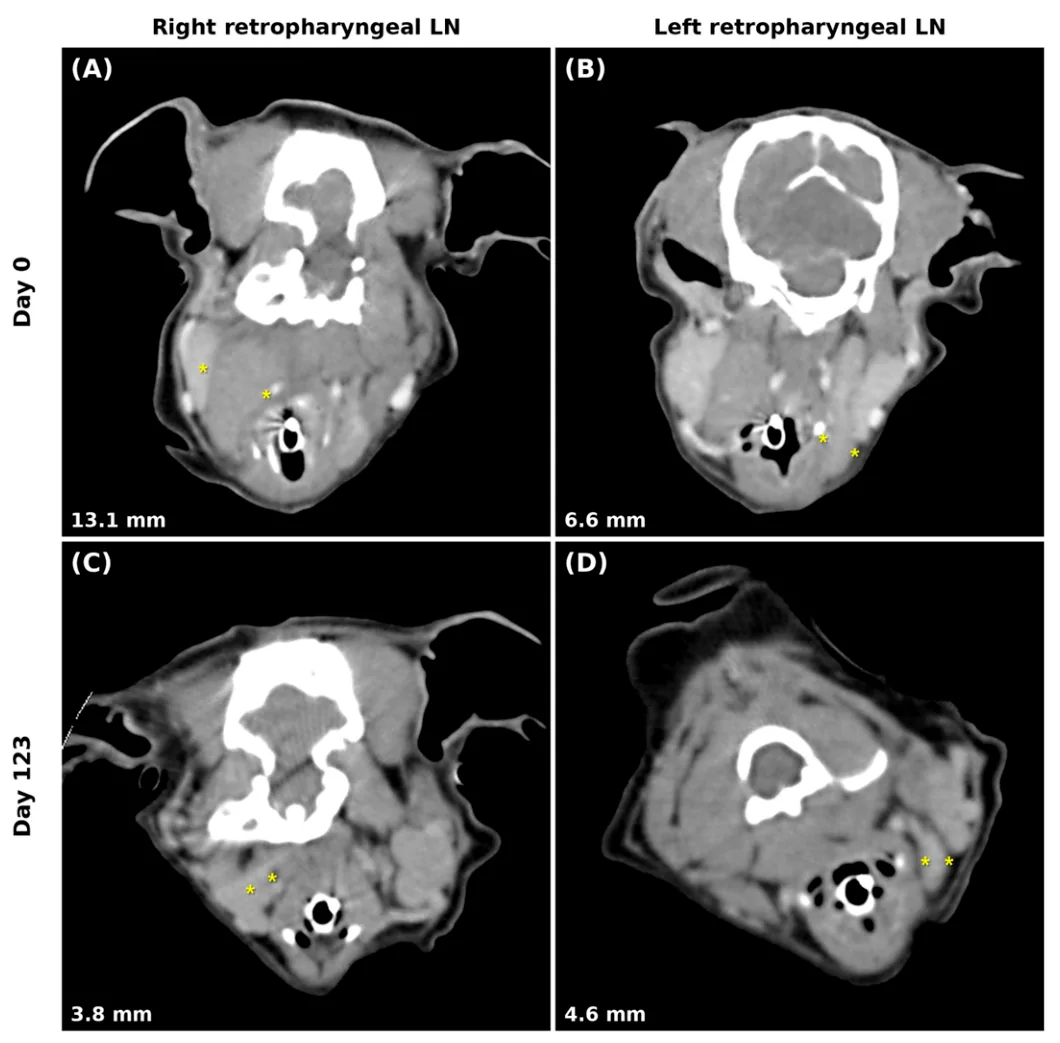
Transverse computed tomographic images of the retropharyngeal lymph nodes. (**A**,**B**) At diagnosis (Day 0), the right retropharyngeal lymph node is enlarged (short axis 13.1 mm; (**A**)), and the left node is markedly elongated (short axis 6.6 mm, long axis 28.5 mm; (**B**)). (**C**,**D**) At restaging after 12 cycles of chemotherapy (Day 123), both nodes are smaller (short axis 3.8 mm on the right, (**C**); 4.6 mm on the left, (**D**)). Asterisks (*) indicate the right (**A**,**C**) and left (**B**,**D**) retropharyngeal lymph nodes.

**Figure 2:**
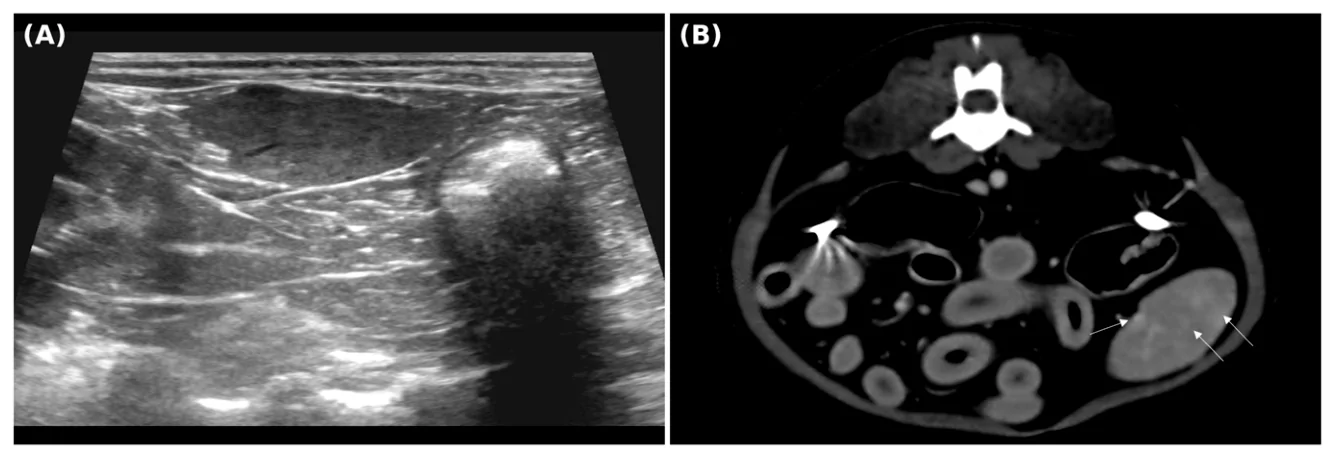
Splenic findings at diagnosis. (**A**) Abdominal ultrasonography showing multifocal hypoechoic lesions in the splenic parenchyma. (**B**) Computed tomography showing splenomegaly with multiple small hyperattenuating nodules (arrows).

The complete blood count and serum calcium concentration were within reference intervals, whereas alanine aminotransferase (ALT) and alkaline phosphatase (ALP) activities were increased (Table 2). Computed tomography (CT) showed enlargement of the bilateral mandibular, retropharyngeal, prescapular, and medial iliac lymph nodes, the hepatic lymph node, and the left internal iliac lymph node (Figure 1A,B), together with splenomegaly and multiple small hyperattenuating nodules in the splenic parenchyma (Figure 2B). Other findings included a nodule protruding from the serosa of the gastric cardia (8.3 × 8.6 mm), a hypoechoic hepatic nodule, and multiple small subcutaneous nodules along the dorsal trunk from the thoracic to the pelvic region. Abdominal ultrasonography confirmed multifocal hypoechoic splenic lesions (Figure 2A).

Cytology of a fine-needle aspirate (FNA) from the right mandibular lymph node showed that more than 90% of the lymphocytes were intermediate to large cells, 1.5–2 times the diameter of an erythrocyte, consistent with high-grade large cell lymphoma. PARR performed on the same smears, however, showed polyclonal rearrangements of both the immunoglobulin heavy chain (IgH) and T-cell receptor gamma (TCRγ) genes.

PARR was performed by a commercial laboratory (CellPurics, Seongnam, Republic of Korea) through a veterinary diagnostic service (GREEN VET, Yongin, Republic of Korea), using DNA extracted from the submitted stained cytological smears. The complementarity-determining region 3 (CDR3) of the IgH and TCRγ genes was amplified by PCR, with amplification of the immunoglobulin mu constant region (Cμ) as a positive control for DNA quality and a distilled water negative control included in each run. The products were separated by capillary electrophoresis on an automated nucleic acid analyzer using a cartridge containing a DNA size marker, and peaks and fragment sizes were analyzed with the analyzer software. A single dominant peak or two peaks within a narrow size range was interpreted as monoclonal, and a broad, Gaussian-like distribution of peaks as polyclonal; a result showing both features was reported as mixed monoclonal and polyclonal. The electropherograms of all three samples are provided in Figure S1.

### 2.3. Histopathological Diagnosis and Staging

**Figure 3:**
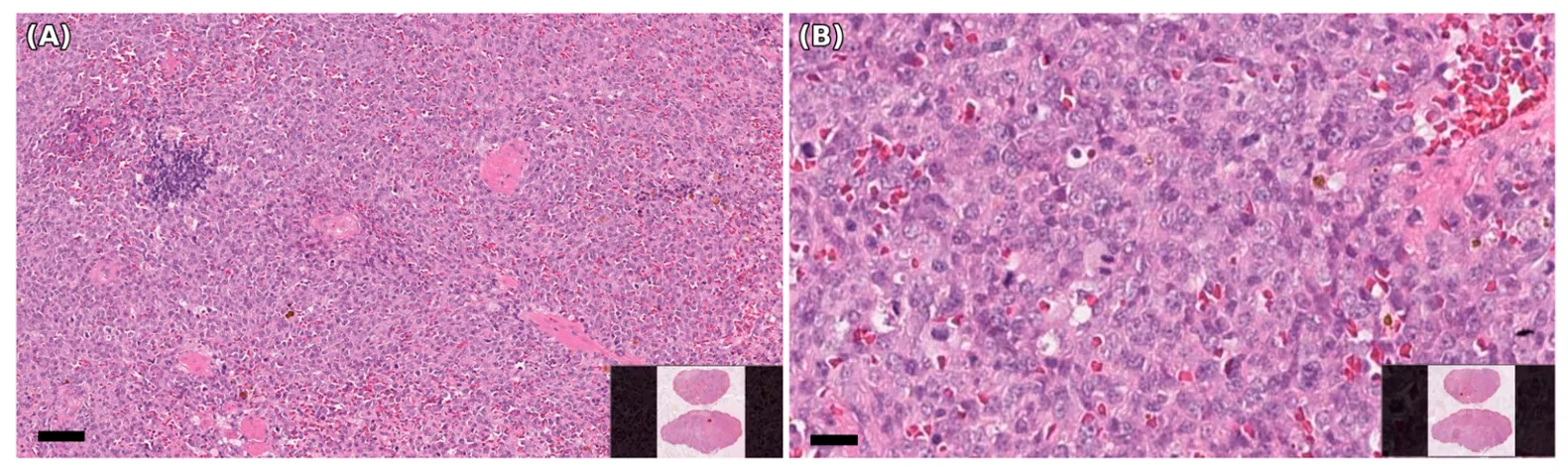
Histopathology of the spleen (hematoxylin and eosin). (**A**) Low magnification showing expansion of the splenic parenchyma by a monotonous population of neoplastic lymphocytes. (**B**) High magnification showing a monotonous population of intermediate to large lymphocytes with prominent nucleoli and cellular atypia. Scale bars: (**A**) 100 µm; (**B**) 20 µm.

As cytology and PARR were discordant, tissue was needed for a definitive diagnosis. Excisional biopsy of a peripheral lymph node was considered. However, the spleen was enlarged and diffusely involved by multiple nodules, and splenectomy was therefore chosen to obtain a larger and more representative tissue sample while removing the enlarged organ. It was performed on Day 10 after the anesthetic and surgical risks had been discussed with the owner, and surgery and recovery were uneventful. On histopathology, the splenic parenchyma was expanded by enlarged, partly coalescing lymphoid nodules composed of a monotonous population of intermediate to large lymphocytes. Their nuclei were more than twice the diameter of an erythrocyte, with one or more prominent nucleoli and marked atypia, and 27 mitoses were counted in 10 high-power fields (Figure 3). A diagnosis of splenic lymphoma, large cell type, was made. The high mitotic count indicated a high-grade lymphoma. The pathologist recommended immunohistochemistry to determine the subtype [11]; however, it was not performed at the time, and no archived tissue remained for subsequent testing. On the basis of splenic involvement, the disease was assigned to World Health Organization clinical stage IV [12]; stage V could not be excluded because the bone marrow and peripheral blood were not evaluated. In the absence of systemic signs, substage a was assigned.

### 2.4. Treatment and Response

After histological confirmation, a CHOP-based protocol consisting of vincristine, cyclophosphamide, doxorubicin, and prednisolone was started on Day 13 and given at approximately weekly intervals for 12 cycles (Table 3). Prednisolone was administered orally and tapered according to the protocol. The first dose of doxorubicin was given at 1 mg/kg. Considering the cardiotoxic potential of the drug [13] and the pre-existing cardiac disease, the second and third doxorubicin doses were reduced by 25% to 0.75 mg/kg. During induction, the dog was hospitalized between Days 19 and 24 for suspected pancreatitis and recovered with supportive care. The remaining cycles were completed without major adverse events.

Restaging CT was performed on Day 123, 2 weeks after the final treatment, and all previously enlarged lymph nodes had decreased in size (Table 4). The retropharyngeal lymph nodes, which were the largest at baseline, have an oblique and elongated orientation that makes long-axis measurement on transverse images unreliable, so their short-axis diameter was also recorded [14]. The short-axis diameter had decreased from 6.6 to 4.6 mm on the left and from 13.1 to 3.8 mm on the right (Figure 1C,D), and the larger of the two left mandibular lymph nodes could no longer be identified. The gastric nodule (9.2 × 9.4 mm) and the subcutaneous nodules were unchanged and were not sampled. When the Veterinary Cooperative Oncology Group (VCOG) response criteria were applied to the CT measurements [15], all measurable lymph nodes had decreased markedly. Because the non-nodal lesions persisted and were not examined, a complete response could not be confirmed, and the findings were interpreted as a marked nodal response.

Echocardiography on the same day revealed thickening and moderate prolapse of the anterior mitral valve leaflet with mitral regurgitation. Left atrial and left ventricular dimensions were within normal limits (left atrium-to-aorta ratio 1.35; normalized left ventricular internal diameter in diastole 1.3), corresponding to American College of Veterinary Internal Medicine (ACVIM) stage B1 myxomatous mitral valve disease (MMVD) with grade 1 diastolic dysfunction [16]. This was the first echocardiographic evaluation at the authors’ hospital, and the cardiac medication was continued. The restaging CT also showed progression of atelectasis or bronchopneumonia in the right cranial and accessory lung lobes, with volume loss and ground-glass opacity.

After completion of chemotherapy on Day 109, oral prednisolone was continued at 0.1 mg/kg twice daily because of intermittent coughing and radiographic evidence of bronchial inflammation. Follow-up examinations on Days 139, 164, and 171 included physical examination and blood tests, with thoracic radiography on Day 171 because of coughing; no peripheral lymph node enlargement was detected on palpation, and no other clinically significant abnormalities were recorded. No abdominal or lymph node imaging was performed between Day 123 and Day 181.

### 2.5. Vulvar Swelling and Disease Progression

**Figure 4:**
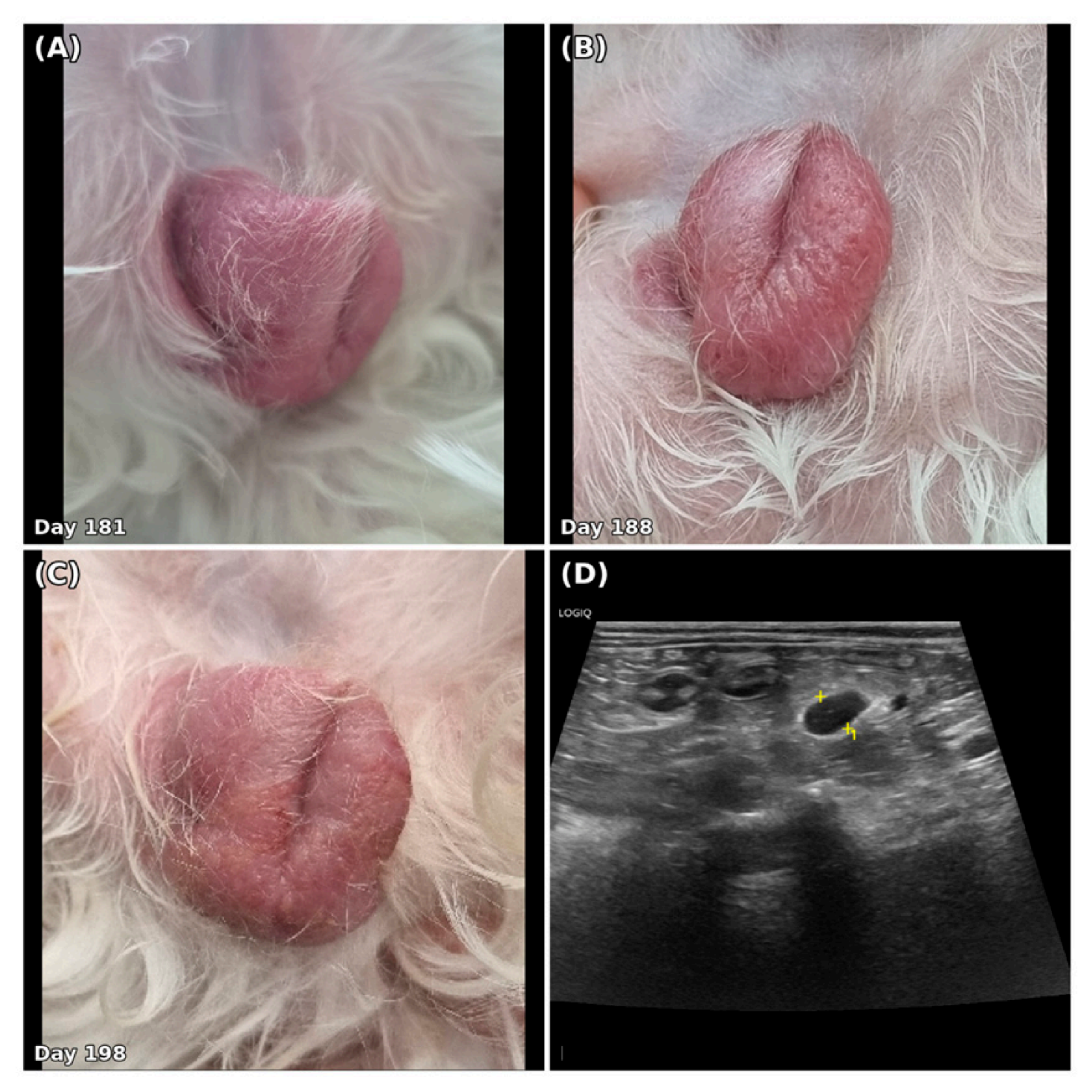
Vulvar swelling and regional lymphadenopathy at disease progression. (**A**) Day 181, when the swelling was first noticed. (**B**) Day 188, showing progression despite antimicrobial and anti-inflammatory treatment. (**C**) Day 198, at the time of fine-needle aspiration. (**D**) Ultrasonographic image of an enlarged medial iliac lymph node; the electronic calipers (+) mark the node, which measured 4.83 mm. The number "1" is an automatic caliper label generated by the ultrasound unit during measurement and appears in the original image.

On Day 181, 168 days after the start of chemotherapy, the owner noticed swelling of the vulva with no history of trauma (Figure 4A). The swelling progressed despite treatment with oral amoxicillin–clavulanic acid (12.5 mg/kg twice daily) from Day 181 (Figure 4B). From Day 184, the prednisolone dose was increased to 0.3 mg/kg orally twice daily, and a topical ointment containing betamethasone dipropionate, clotrimazole, and gentamicin sulfate was applied, without apparent effect. Vaginoscopy on Day 188 revealed no inflammatory or mass lesions within the vaginal lumen, and the lesion was considered to be confined to the vulva. No vaginal discharge or behavioral signs of estrus were observed, and ovarian remnant syndrome was therefore considered unlikely; however, vaginal cytology and hormonal testing were not performed. Serum albumin was within the reference interval (Table 2). Bacterial and fungal cultures of urine collected by cystocentesis yielded no growth, and serum canine C-reactive protein (c-CRP) remained within the reference interval (Table 2). Abdominal ultrasonography on Day 188 showed enlargement of the medial iliac lymph node (short axis 4.83 mm; Figure 4D), and on Day 192 both superficial inguinal lymph nodes were enlarged (short axis 8.05 mm on the left and 6.91 mm on the right), exceeding the mean values reported for presumptively normal dogs [17]. These ultrasonographic measurements are not directly comparable with the CT measurements obtained at restaging, and the reference values were derived from considerably larger dogs. The prescapular lymph nodes had also enlarged compared with the restaging CT. The sonographer considered microscopic infiltration by lymphoma and lymphatic stasis secondary to regional lymphadenopathy as possible causes of the vulvar swelling.

**Figure 5:**
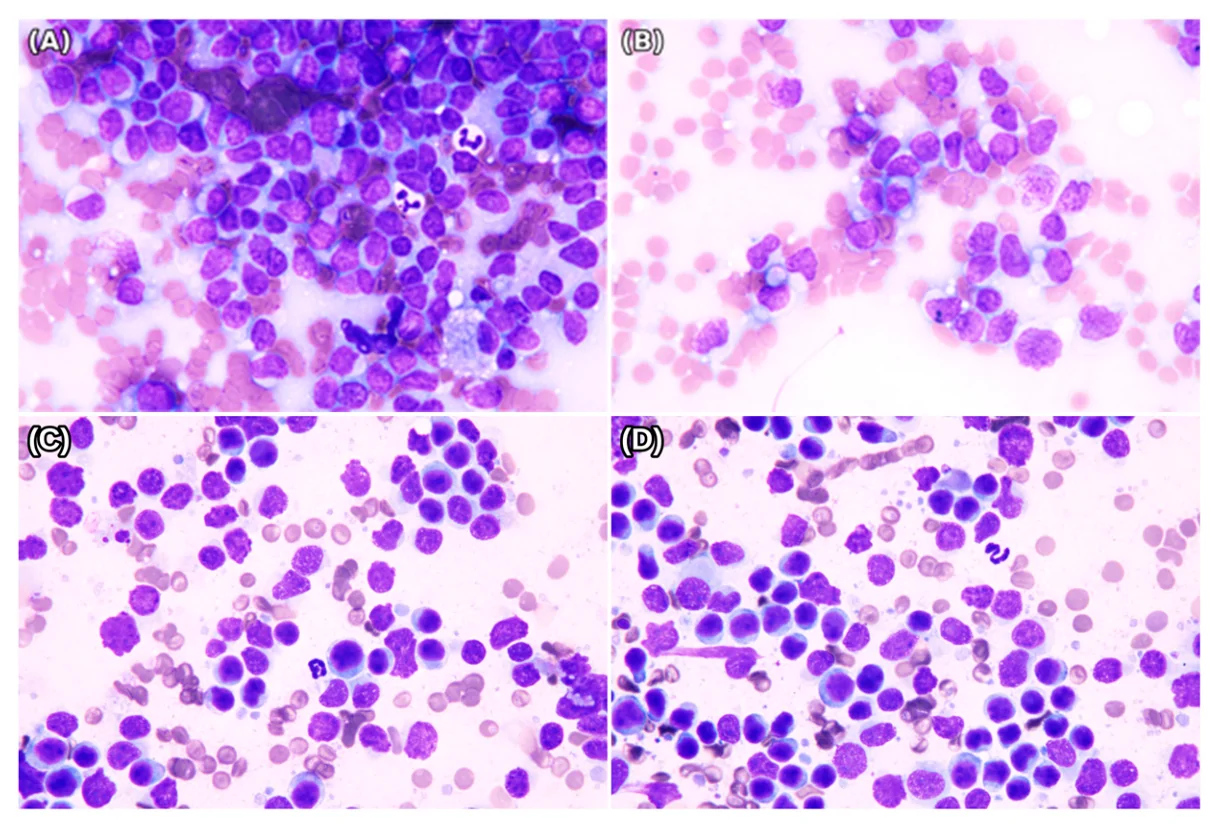
Cytology of fine-needle aspirates (Diff-Quik). (**A**,**B**) Vulva (Day 198), showing a predominance of intermediate to large lymphocytes with mild atypia, compatible with intermediate to large cell lymphoma. (**C**,**D**) Inguinal lymph node (Day 192), showing numerous intermediate to large lymphocytes with fewer small lymphocytes.

FNA of the inguinal lymph nodes was performed on Day 192, with aspirates obtained from two right and one left inguinal lymph node. Separate smears prepared from the same aspirates were reviewed in-house and submitted to the reference laboratory. The reference laboratory reported that more than 50% of the cells were intermediate to large lymphocytes with multiple prominent nucleoli and coarse chromatin, with fewer small lymphocytes, supporting a diagnosis of large cell lymphoma (Figure 5C,D). The in-house review by the attending clinician, which was not performed by a clinical pathologist, had suggested a higher proportion of small lymphocytes; the diagnosis was based on the reference laboratory report. On the basis of this report, disease progression was diagnosed on Day 198, and rescue treatment with vincristine (0.7 mg/m^2^) was started. An FNA of the swollen vulva was obtained on the same day (Figure 4C).

### 2.6. Outcome

Shortly after vincristine administration, the dog developed cyanosis and acute dyspnea and was hospitalized. Thoracic radiographs showed an infiltrate in the right caudal lung lobe, and diuretic treatment was started. On Day 199, blood urea nitrogen, creatinine, and phosphorus concentrations had increased compared with the values before progression (Table 2); potassium, calcium, and uric acid were not measured. Mild respiratory discomfort persisted on Day 199, but no further deterioration was observed overnight. On the morning of Day 200, however, the dog suffered sudden cardiopulmonary arrest and did not respond to cardiopulmonary resuscitation. A necropsy was not performed.

The results of the samples obtained at progression became available after death. PARR of the inguinal lymph node aspirate showed a mixed monoclonal and polyclonal IgH pattern with a polyclonal TCRγ pattern. Cytology of the vulvar aspirate, which was of moderate cellularity, showed that more than 50% of the cells were intermediate to large lymphocytes with mild atypia, multiple prominent nucleoli, and occasional mitotic figures, consistent with intermediate to large cell lymphoma (Figure 5A,B). PARR of this sample revealed a clonal IgH rearrangement with a polyclonal TCRγ pattern, suggestive of, but not establishing, a B-cell origin (Table 5). These findings were cytologically suggestive of lymphomatous infiltration of the vulva, although histopathological confirmation was not obtained.

## 3. Discussion

### 3.1. Clinical Significance

In this dog with multicentric lymphoma and splenic involvement, vulvar swelling was the first sign of disease progression noticed after a marked nodal response to chemotherapy. Two aspects of the case are of clinical interest: the unusual site of progression and the variation in PARR results between samples.

Lymphoma of the canine female reproductive tract is rare. The cases reported so far involved the uterus or ovaries and were diagnosed after ovariohysterectomy for suspected pyometra [3,4]. Lesions at mucocutaneous junctions, including the vulva, are common in cutaneous epitheliotropic T-cell lymphoma [5,6], but that disease is a primary cutaneous T-cell neoplasm. In the present dog, the vulvar lesion appeared after a nodal response of a multicentric lymphoma and carried a clonal IgH rearrangement. Because the regional lymph nodes were enlarged at the same time, lymphatic stasis may have contributed to the swelling, as the sonographer suggested. Nevertheless, the cytological findings and the clonal IgH rearrangement in the vulvar aspirate suggested infiltration of the tissue by lymphoma, although histological confirmation was not obtained. The subcutaneous nodules present at diagnosis were never sampled, so cutaneous involvement from the outset cannot be excluded. Moreover, as the non-nodal lesions persisted and no imaging was performed between Day 123 and Day 181, the vulvar lesion may represent progression of residual disease rather than a new relapse, and earlier nodal enlargement may have gone unnoticed. In practice, vulvar swelling that does not respond to antimicrobial and anti-inflammatory treatment in a dog with a history of lymphoma should prompt early cytological evaluation. In this dog, a c-CRP concentration within the reference interval during progression of the swelling and a negative urine culture also argued against an infectious cause. Because immunophenotyping was not performed and PARR does not reliably establish lineage [10], the B-cell origin of both lesions remains presumptive.

At the time of progression, ALP activity had risen to 3770 U/L and the white blood cell count to approximately 31 K/µL (Table 2). These changes may have reflected corticosteroid administration, including the increase in prednisolone to 0.3 mg/kg twice daily and the concurrent topical corticosteroid from Day 184, together with inflammation associated with the vulvar lesion. Hepatic infiltration could not be excluded because the hepatic nodule was never sampled, and bone marrow and peripheral blood were not evaluated for neoplastic cells.

### 3.2. Interpretation of Discordant PARR Results

The PARR results differed among the three samples (Table 5). Because the samples were obtained from different sites at different time points, before and after chemotherapy and during systemic and topical corticosteroid administration, spatial and temporal factors cannot be separated, and the discrepancies may have more than one explanation. At diagnosis, more than 90% of the lymphocytes in the mandibular lymph node aspirate were intermediate to large cells, which makes dilution of a clonal signal by reactive lymphocytes less likely. Failure of the consensus primers to amplify the rearranged gene of the neoplastic clone, for example because of somatic hypermutation, is one possible explanation [8,9,10]; however, this could not be verified, and limited DNA yield or quality from stained smears, or misinterpretation of the cytological findings, cannot be excluded.

At progression, the reference laboratory described a similar cytological composition in the inguinal lymph node and vulvar aspirates, with more than 50% intermediate to large lymphocytes in both, yet their IgH results differed. On the basis of these reports, the difference cannot be readily attributed to the proportion of neoplastic cells. Notably, the inguinal lymph node sample also showed a prominent dominant IgH peak, differing from the vulvar sample mainly in the number of accompanying smaller peaks (Figure S1C,E). Mixed patterns of this type are classified as equivocal, as they may represent a small neoplastic clone among reactive lymphocytes or antigen-driven expansion, and repeat sampling is recommended [9]. The difference between the in-house and reference laboratory assessments of separate smears from the same inguinal aspirates suggests that cellular composition may vary among smears, and the smears used for PARR may not have been fully representative; differences in DNA yield or quality and concurrent corticosteroid administration are other possible contributors. Conversely, poorly cellular samples can yield a spurious dominant peak [10]; although the vulvar aspirate was of moderate cellularity and its cytological features supported lymphoma, replicate testing was not reported. Furthermore, a clonal result in the vulvar lesion does not show that it arose from the same clone as the original tumor. On the electropherograms, the dominant IgH peaks of the inguinal lymph node and vulvar samples appeared at different positions (Figure S1C,E); however, their fragment sizes were not provided by the laboratory, and the clonal relationship between the two lesions could therefore not be assessed. These observations do not establish the mechanism of the discordance, but they illustrate that a single polyclonal or equivocal PARR result should not be used to exclude lymphoma when cytological findings are suggestive [10].

### 3.3. Role of Splenectomy and Assessment of Response

Splenectomy was performed primarily to obtain tissue for diagnosis rather than as a therapeutic procedure. In dogs with lymphoma confined to the spleen, splenectomy has been associated with a favorable outcome, whereas involvement of additional sites carried a significantly poorer prognosis [18]. The present dog had generalized lymphadenopathy at the time of surgery and therefore did not belong to that group, and systemic chemotherapy was required. Splenectomy in a small dog with cardiac and airway disease carries anesthetic and surgical risk, and excision of a peripheral lymph node would have been less invasive. In this case, the enlarged and diffusely involved spleen was considered to provide the most representative tissue, and the procedure was completed without complications.

The nodal response to CHOP and the time to progression were within the range of published data. In 134 dogs treated with a CHOP protocol, the median progression-free survival measured from the start of treatment was 176 days overall and 202 days in dogs that achieved a complete response [19]. Calculated in the same way, progression-free survival in the present dog was 168 days. The distinctive feature of this case was therefore the manner of progression rather than its timing. As the gastric and subcutaneous nodules persisted and were not examined, the response was assessed on lymph node measurements alone.

### 3.4. Considerations Regarding the Cause of Death

Without a necropsy, the cause of death could not be determined, and the following considerations are speculative. Cardiac decompensation cannot be excluded: cardiovascular disease has been associated with shorter survival in small breed dogs treated with CHOP [20], and the most recent echocardiography had been performed approximately 2.5 months earlier, although it showed stage B1 MMVD without chamber enlargement at that time [16]. The cumulative doxorubicin dose of approximately 34 mg/m^2^ was well below the lifetime limit commonly applied in dogs [13,19]. Pre-existing pulmonary changes, a new infiltrate in the right caudal lobe, and the abrupt clinical course would also be compatible with pneumonia, pulmonary infiltration by lymphoma, or pulmonary thromboembolism, the last of which is associated with neoplasia in dogs [21,22]. The increases in blood urea nitrogen and phosphorus after rescue vincristine may have reflected prerenal azotemia related to diuretic therapy; creatinine remained within the reference interval, and acute tumor lysis syndrome, although reported in dogs with lymphoma after chemotherapy [23,24], could not be evaluated because potassium, calcium, and uric acid were not measured.

### 3.5. Limitations

This report has several limitations. No necropsy and no biopsy of the vulvar lesion were performed. Immunohistochemistry was not carried out at diagnosis, and no archived tissue remained for subsequent analysis; the immunophenotype of the original tumor and its clonal relationship with the later lesions therefore remain unknown. The gastric, hepatic, and subcutaneous nodules were not sampled, so a complete response could not be confirmed, and the vulvar lesion may represent progression of residual disease. No abdominal or lymph node imaging was performed between Day 123 and Day 181. Low-dose prednisolone was continued after chemotherapy for bronchial disease and was increased during the period of vulvar swelling, which may have influenced the timing of clinical progression and the cytological and clonality findings. Vaginal cytology and hormonal testing for ovarian remnant syndrome were not performed. PARR was performed by a commercial laboratory; the primer sequences were not disclosed, and replicate testing was not reported. Response was assessed by applying VCOG criteria, designed for caliper measurement of peripheral lymph nodes, to CT measurements; ultrasonographic and CT measurements were compared; and the reference values used were derived from considerably larger dogs [14,15,17]. The vulvar cytology and both PARR results obtained at progression were reported after death. Finally, electrolytes, uric acid, and coagulation parameters were not measured before death.

## 4. Conclusions

In dogs treated for multicentric lymphoma, vulvar swelling that fails to respond to antimicrobial or anti-inflammatory treatment may reflect neoplastic infiltration and should prompt early cytological and, where possible, histological evaluation. PARR results may differ between samples obtained at different sites and time points in the same patient, and a polyclonal or equivocal result should be interpreted together with cytology and, ideally, immunophenotyping rather than used alone to exclude lymphoma.

## Figures and Tables

**Table 1:** Timeline of the main diagnostic and therapeutic events.

Day	Event
0	Presentation with generalized lymphadenopathy; CT; FNA cytology and PARR of mandibular lymph node
10	Splenectomy; histopathological diagnosis of large cell lymphoma
13	Start of CHOP-based chemotherapy
19–24	Hospitalization for suspected pancreatitis
109	Twelfth and final chemotherapy treatment
123	Restaging CT and echocardiography; marked nodal response
139, 164, 171	Follow-up examinations and blood tests; thoracic radiography on Day 171
181	Vulvar swelling first noticed by the owner
188	Vaginoscopy and abdominal ultrasonography
192	FNA cytology and PARR of inguinal lymph nodes
198	Diagnosis of disease progression; rescue vincristine; FNA of the vulva; acute dyspnea
200	Cardiopulmonary arrest and death

**Table 2:** Selected hematological and biochemical findings at diagnosis, restaging, and disease progression.

Parameter	Reference Range	Day 0	Day 123	Day 184	Day 198	Day 199
WBC (K/µL)	5.05–16.76	13.10	15.30	27.15	31.54	—
Hematocrit (%)	37.3–61.7	51.6	38.3	37.0	36.2	—
Platelets (K/µL)	148–484	514	932	635	673	—
ALT (U/L)	10–102	374	150	110	340	—
ALP (U/L)	40–312	1012	1129	865	3770	—
Albumin (g/dL)	2.3–4.0	3.4	3.5	3.3	—	—
BUN (mg/dL)	6–25	10.7	27.0	25.1	—	43.9
Creatinine (mg/dL)	0.4–1.8	0.49	0.52	0.66	—	1.16
Calcium (mg/dL)	7.9–12.0	10.7	10.1	8.6	—	—
Phosphorus (mg/dL)	2.5–6.8	3.3	3.0	5.5	—	12.4
c-CRP (mg/L)	0–9	<9	<9	<9	<9	20

Day 0, diagnosis; Day 123, restaging after chemotherapy; Day 184, during progression of vulvar swelling; Day 198, diagnosis of disease progression and rescue treatment; Day 199, one day after rescue treatment. —, not measured. ALP, alkaline phosphatase; ALT, alanine aminotransferase; BUN, blood urea nitrogen; c-CRP, canine C-reactive protein; WBC, white blood cells.

**Table 3:** Chemotherapy protocol administered to the dog.

Cycle	Day	Drug	Dose
1	13	Vincristine	0.5 mg/m^2^
2	23	Cyclophosphamide	200 mg/m^2^
3	30	Vincristine	0.5 mg/m^2^
4	39	Doxorubicin	1 mg/kg
5	48	Vincristine	0.7 mg/m^2^
6	60	Cyclophosphamide	200 mg/m^2^
7	67	Vincristine	0.7 mg/m^2^
8	76	Doxorubicin	0.75 mg/kg (25% reduction)
9	88	Vincristine	0.7 mg/m^2^
10	95	Cyclophosphamide	200 mg/m^2^
11	102	Vincristine	0.5 mg/m^2^
12	109	Doxorubicin	0.75 mg/kg (25% reduction)

Treatments were given at approximately weekly intervals. Prednisolone was administered orally and tapered according to the protocol. The cumulative doxorubicin dose was approximately 34 mg/m^2^.

**Table 4:** Lymph node dimensions measured on computed tomography at diagnosis (Day 0) and at restaging (Day 123).

Lymph Node	Day 0 Short/Long Axis (mm)	Day 123 Short/Long Axis (mm)
Mandibular, left	5.4/11.2	Not identified
Mandibular, right	11.1/15.2	2.1/2.8
Retropharyngeal, left	6.6/28.5	4.6/13.5
Retropharyngeal, right	13.1/23.6	3.8/9.2
Prescapular, left	10.1/10.7	4.6/6.2
Prescapular, right	12.2/15.6	3.5/5.1
Hepatic	5.9/11.4	3.7/5.2
Medial iliac, left	3.0/4.4	1.7/2.5
Medial iliac, right	5.4/6.0	1.9/2.2
Internal iliac, left	3.7/4.3	1.6/1.6

For the mandibular lymph nodes, two nodes were identified on each side, and the larger node is shown. Because of their oblique and elongated orientation, long-axis measurement of the retropharyngeal lymph nodes on transverse images is unreliable, and the short-axis diameter is also presented [14]. The gastric nodule (9.2 × 9.4 mm) and the subcutaneous nodules were unchanged at restaging.

**Table 5:** Cytological and PARR results according to the time and site of sampling.

Day	Sampling Site	Cytology	PARR (IgH)	PARR (TCRγ)	Result Reported
Day 0	Mandibular lymph node	Large cell lymphoma (>90% intermediate to large lymphocytes)	Polyclonal	Polyclonal	Day 11
Day 192	Inguinal lymph node	Large cell lymphoma (>50% intermediate to large lymphocytes, reference laboratory); higher proportion of small lymphocytes suggested on in-house review of separate smears	Mixed monoclonal and polyclonal	Polyclonal	After death
Day 198	Vulva	Intermediate to large cell lymphoma	Monoclonal	Polyclonal	After death

IgH, immunoglobulin heavy chain; PARR, PCR for antigen receptor rearrangement; TCRγ, T-cell receptor gamma. Electropherograms are shown in Figure S1.

## Data Availability

The original contributions presented in this study are included in the article/Supplementary Material. Further inquiries can be directed to the corresponding author.

## Supplementary Materials

The following supporting information can be downloaded at: https://www.mdpi.com/article/10.3390/vetsci13101102/s1, Figure S1: PARR electropherograms of the immunoglobulin heavy chain (IgH) and T-cell receptor gamma (TCRγ) genes in samples obtained at diagnosis and at disease progression.

## Abbreviations

The following abbreviations are used in this manuscript:

ACVIM	American College of Veterinary Internal Medicine
ALP	Alkaline phosphatase
ALT	Alanine aminotransferase
BUN	Blood urea nitrogen
c-CRP	Canine C-reactive protein
CDR3	Complementarity-determining region 3
CHOP	Cyclophosphamide, doxorubicin, vincristine, and prednisolone
CT	Computed tomography
FNA	Fine-needle aspirate
IgH	Immunoglobulin heavy chain
LN	Lymph node
MMVD	Myxomatous mitral valve disease
PARR	PCR for antigen receptor rearrangement
PCR	Polymerase chain reaction
TCRγ	T-cell receptor gamma
VCOG	Veterinary Cooperative Oncology Group
