# Supplementary material for: Cytologically Suggested Vulvar Involvement in a Dog with Multicentric Lymphoma After a Nodal Response to CHOP-Based Chemotherapy, with Discordant Clonality Results: A Case Report
Source: Vet Sci. 2026 Oct 16;13(10):1102. doi: 10.3390/vetsci13101102 (PMC13709344; doi:10.3390/vetsci13101102)
Supplement: Supplementary file 1 [file vetsci-13-01102-s001.zip › vetsci-4613961-supplementary.pdf]

Supplementary Materials

Cytologically Suggested Vulvar Involvement in a Dog with Multicentric Lymphoma After a Nodal Response to CHOP-Based Chemotherapy, with Discordant Clonality Results: A Case Report

Taehyun Roh and Kunho Song

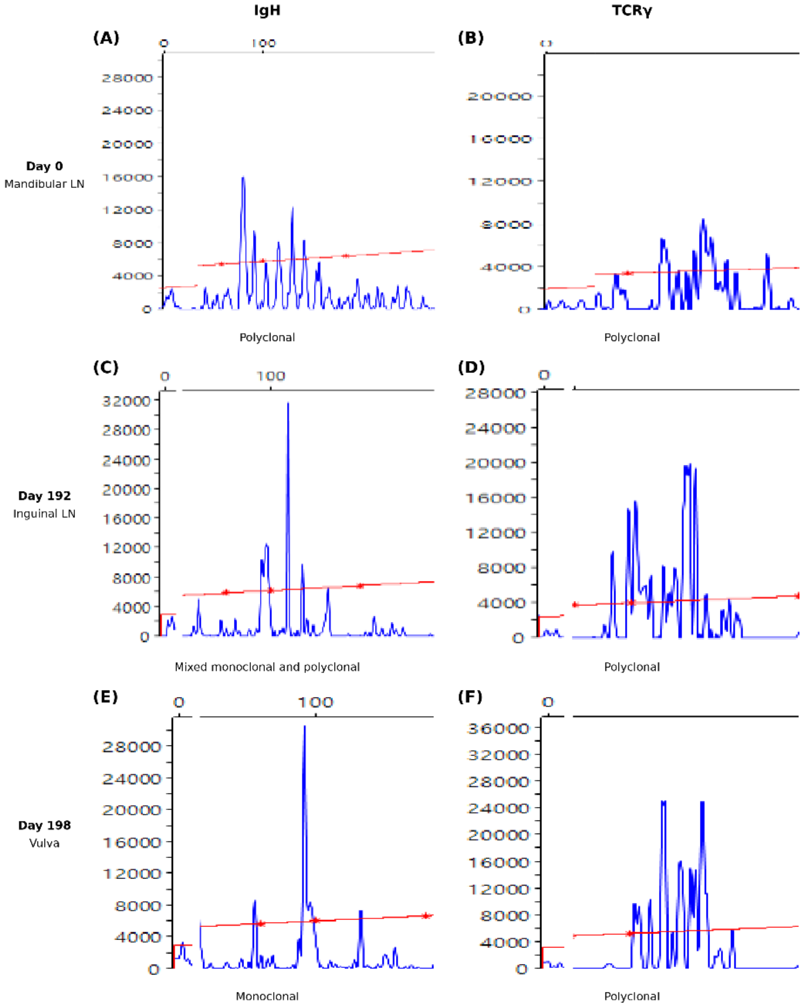

Figure S1. PARR electropherograms of the immunoglobulin heavy chain (IgH) and T-cell receptor gamma (TCRγ) genes in samples obtained at diagnosis and at disease progression. (A,B) Mandibular lymph node at diagnosis (Day 0): polyclonal IgH and TCRγ patterns. (C,D) Inguinal lymph node at disease progression (Day 192): mixed monoclonal and polyclonal IgH pattern and polyclonal TCRγ pattern. (E,F) Vulvar aspirate (Day 198): monoclonal IgH and polyclonal TCRγ patterns. The electropherograms are reproduced from the laboratory reports without modification, and their resolution is limited by that of the original images. PARR, polymerase chain reaction for antigen receptor rearrangement.
